# A Review on Data Fusion of Multidimensional Medical and Biomedical Data

**DOI:** 10.3390/molecules27217448

**Published:** 2022-11-02

**Authors:** Kazi Sultana Farhana Azam, Oleg Ryabchykov, Thomas Bocklitz

**Affiliations:** 1 Leibniz Institute of Photonic Technology, Member of Leibniz-Research Alliance “Health Technologies”, Albert-Einstein-Straße 9, 07745 Jena, Germany; 2 Institute of Physical Chemistry and Abbe Center of Photonics, Friedrich Schiller University Jena, Helmholtzweg 4, 07743 Jena, Germany

**Keywords:** data fusion, ultrasonography, single photon emission computed tomography, positron emission tomography, magnetic resonance imaging, computed tomography, Raman spectroscopy, MALDI imaging, mammography, fluorescence lifetime imaging microscopy, deep learning, machine learning

## Abstract

Data fusion aims to provide a more accurate description of a sample than any one source of data alone. At the same time, data fusion minimizes the uncertainty of the results by combining data from multiple sources. Both aim to improve the characterization of samples and might improve clinical diagnosis and prognosis. In this paper, we present an overview of the advances achieved over the last decades in data fusion approaches in the context of the medical and biomedical fields. We collected approaches for interpreting multiple sources of data in different combinations: image to image, image to biomarker, spectra to image, spectra to spectra, spectra to biomarker, and others. We found that the most prevalent combination is the image-to-image fusion and that most data fusion approaches were applied together with deep learning or machine learning methods.

## 1. Introduction

Data fusion refers to the combined analysis of data sets that contain multiple types of data. The main reason for this approach is a comprehensive characterization of the sample, which is often not possible with a single data type or a single analytical method alone. For example, malignant tumors are difficult to detect with one measurement method, e.g., data type, due to various reasons such as low specificity and low benign predictability [1]. So, data fusion makes it possible to obtain extended information about a sample, leading to a better prediction, e.g., to a more accurate diagnosis of a patient. This review summarizes different data fusion schemes based on deep learning and classical machine learning approaches over multiple measurement techniques including ultrasonography, single photon emission computed tomography, positron emission tomography, magnetic resonance imaging, computed tomography, Raman spectroscopy, MALDI imaging, mammography, and fluorescence lifetime imaging microscopy.

Generally, data fusion can be classified into three categories: pixel-level, feature-level, and decision-level fusion. Pixel-level fusion is a direct process that combines the data from multiple sources. Fusion at the feature level utilizes machine learning and statistical approaches for combining the features extracted from different data types. The combination of variables, which are associated with decision rules, is referred to as decision-level fusion. Typically, the data fusion process of biomedical data involves a number of preprocessing steps, such as temporal and spatial alignment, noise and background removal, dimensionality reduction, and handling of missing data [1]. 

Various algorithms are employed in biomedical image fusion experiments resulting in an improved prediction of properties of biomedical specimen, thereby the enhanced effectiveness of clinical functions are demonstrated [2]. In pixel-level fusion, the pixels of the images should overlap with each other and carry information about the same sample regions. For example, microscopic and macroscopic images can be fused, whereas the combination between ultrasound images and a mammogram is more problematic because it is impossible to co-register pixels. This results from a highly different scale and field of view of both imaging techniques. The process of feature fusion entails extracting features from different image modalities before performing the fusion itself. Feature-level fusion reduces data preprocessing, including the alignment of the image data. The methods used for traditional data fusion are fuzzy logic, wavelet transforms, neural networks, support vector machines, and morphological approaches [2]. Besides these classical approaches, deep learning methods with multiple inputs can be used that fuse features in the network. 

**Figure 1:**
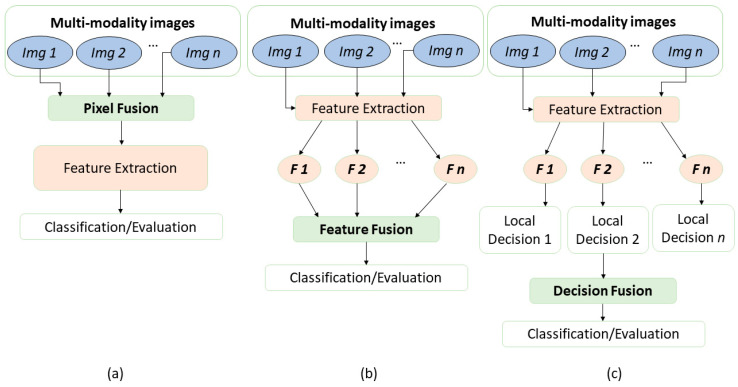
An overview of image fusion levels. The diagram describes three levels of data fusion to classify or evaluate the extracted information of combined images. (**a**) represents pixel-level fusion, (**b**) represents the feature-level fusion, and (**c**) corresponds to decision-level fusion. All data fusion schemes aim to use the information from all modalities to make the decision more accurate than the decision achieved with a single modality alone. Adapted with permission from reference [3], 2021, Elsevier.

Combining data on high-level, or decision-level fusion also requires feature extraction. From the extracted features, the local-level decisions/predictions are made. In the decision-level fusion, these decisions/predictions are fused to form a decision/prediction for the fused data. Here, an overview of image fusion can be found in Figure 1.

There are three main sections in this review paper. A brief introduction of data fusion and different data fusion methods are offered in Section 1. Multiple data types including ultrasonography, single photon emission computed tomography, positron emission tomography, magnetic resonance imaging, computed tomography, Raman spectroscopy, MALDI imaging, mammography, and fluorescence lifetime imaging spectroscopy are widely used for the data fusion technique. An overview of these different data types is covered in Section 2. Lastly, the techniques and algorithms based on deep learning and machine learning as well as the combination of multimodal data: image, spectra, biomarkers, etc. are discussed in Section 3.

## 2. Overview of Data Types

Ultrasonography, single photon emission computed tomography, positron emission tomography, magnetic resonance imaging, computed tomography, Raman spectroscopy, MALDI imaging, mammography, and fluorescence lifetime imaging microscopy are the most popular imaging modalities employed in different organ investigations. These varieties of modalities are used in lung disease investigation, bone marrow inspection, prostate cancer detection, breast area projection for breast cancer, brain imaging conduction [2], and oral cancer identification [4].

From the perspective of application levels, we can group these imaging modalities in different levels: screening, diagnostic, cellular, or molecular. The screening techniques detect diseases earlier and make the monitoring of potential health risks possible. Diagnostic techniques narrow down the diagnosis and help physicians to create the most effective treatment plans that improve patient outcome. Additionally, at the cellular level, the specific responses onto stimuli or local changes are detected, which helps to obtain better understanding of the diseases in the academic research context. In the clinical context, cellular-level techniques make it possible to differentiate specific tumor types or to detect and visualize margins of different tissue types. Selected techniques can even detect changes on a molecular level, going even deeper into the analysis of patient samples. In such categorizations, most techniques can operate in multiple levels. Thus, cellular response is often used as a diagnostic marker and many techniques are used for screening and diagnostic purposes with only minor adjustments. Hence, information, received from different sources at different levels can be fused and play a significant role in academic research and clinical contexts. In general, getting the information on a molecular level makes the techniques and the results more interesting in basic research. So, depending on how expensive and fast the technique is, it can also be transferred to clinical contexts. Additionally, for clinical contexts it is important to have robust and stable techniques. Thus, different types of data can be combined to provide a better characterization of diseases. A brief introduction of all the data types is presented below. 

### 2.1. Ultrasonography

Ultrasonography is a screening level [5] non-invasive, radiation-free, cost-effective, and real-time medical imaging technology that is widely used in gynecology and echocardiography. It is a low-cost imaging technique compared to other medical imaging technologies. Due to the low resolution of images created by reflecting ultrasound waves, ultrasound images are contaminated by numerous types of noise. This negatively affects the tissue features [6]. Several filtering techniques have been used to reduce speckle noise in medical ultrasonography, including median filters, wiener filters, adaptive filtering techniques, and transform-based techniques such as Fourier transform, wavelet transform, and Hilbert transform [7]. 

### 2.2. Single Photon Emission Computed Tomography (SPECT)

SPECT is a noninvasive way of analyzing cerebral blood flow (as an indirect marker of neuronal activity) and can be used for research and clinical diagnosis (monitoring brain and heart diseases and infections) [8]. This technique provides information on the cellular or molecular level [5]. In SPECT imaging, increasing sensitivity without sacrificing image quality is the most challenging problem. In order to improve the resolution and image quality, post-processing techniques must be used to reduce the signal noise [9].

### 2.3. Positron Emission Tomography (PET)

Positron emission tomography, commonly called PET imaging or PET scan, is an incredibly valuable form of nuclear medicine imaging. This type of molecular imaging [5] has a plethora of functions in radiology research for brain diagnostics and treatment. By demonstrating the effects of fixed motion in image restoration and enhancing the design of the detector, a unified approach to lowering limits is commonly used. A unique understanding of the imaging of molecules is often desired as an advantage of positron emission tomography images [10]. 

### 2.4. Computed Tomography (CT)

Computed tomography, also called CT scan, is a popularly used screening technique for obtaining 3D images of body organs [5]. Earlier lung cancer detection is possible by the application of a model based on CT scan images [11]. Image processing techniques for CT scans include median filtering, thresholds, pre-processing, image erosion, and feature abstraction design [11]. It provides a higher level of contrast resolution and is less expensive, but it exposes patients to a higher level of radiation.

### 2.5. White Light Microscopy

Microscopy is progressively used in biological research and clinical practice to achieve information on the cellular and molecular level [12]. The method has the ability to observe a wide range of biological activity in living cells. However, it has a lower resolution and can only be used with the presence of light. In clinical pathology, it is used for imaging large areas rapidly and nondestructively in 3D and core biopsy [13].

### 2.6. Macroscopic Imaging

Digital cameras are commonly used to keep track of samples and make documentation easier. Anyone can easily acquire photos of the suspected body part, which makes this technique the most accessible nowadays. These images, which are non-uniformly lit, are known as macroscopic images [14]. They can be used in both application schemes for screening and diagnostics. These images are helpful in development management because they allow easy comparison when detecting changes in shape, size, or color that indicate the presence of early symptoms of malignancy.

### 2.7. Mammography

Mammography remains the most effective screening [15] test for detecting breast cancer in women [16]. Mammographic images are difficult to work with because of their low signal-to-noise ratio, which is typically 5–6 dB, equating to a noise level of 3–4 gray levels in intensity [16]. It is an effective tool to reduce the risks of undergoing chemotherapy and dying from breast cancer. However, it involves small amounts of radiation and sometimes it leads to overdiagnosis [17].

### 2.8. Magnetic Resonance Imaging (MRI)

MRI is a medical imaging technique that scans the body using radio waves and it is mostly utilized in the field of radiology. This diagnostic technique provides valuable information on the screening level [5] for disease identification. It is an established diagnostic method for detecting cancer, heart disease, bone, and muscular abnormalities. Medical professionals can use MRI technology for the early detection of brain tumors [18]. 

### 2.9. MALDI-IMS

**Figure 2:**
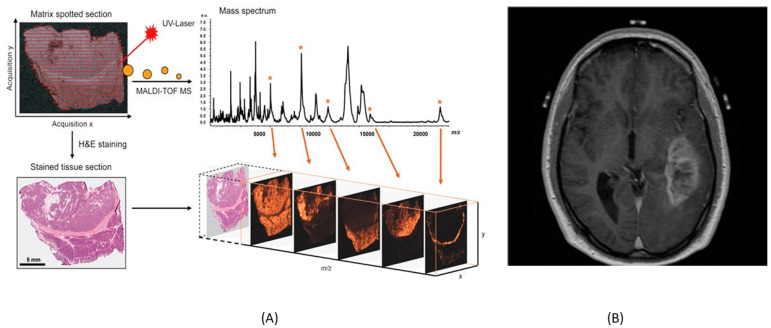
(**A**) MALDI-IMS generating mass spectrum and stained tissue selection with the patches. (**B**) MRI image of brain to detect the diseases using a power full magnetic field and pulse of radiofrequency. (**A**) is reprinted with permission from reference [21], 2015, Springer Nature. (**B**) is reprinted with permission from reference [22], 2020, AIP Publishing.

Matrix-assisted laser desorption/ionization imaging mass spectrometry (MALDI-IMS) is an emerging analytical technique. The technique is based on the ionization of sample molecules and enables the fast detection of various biomolecular species in both biofluids and tissues [19]. The ability to correlate molecular data with conventional histology containing the information of the spatial area of analytes after the mass spectrometric measurement is a significant advantage of MALDI-IMS. It can also be used to distinguish between cancers of different subtypes, stages, or degrees of metastasis, which is important for developing a personalized, individually designed treatment regimen [19]. In clinical contexts, MALDI is used for bacteria classification [20]. Although MALDI-IMS is more commonly used in basic research, it also has a potential for clinical application [21]. Moreover, Figure 2 shows mass spectrum generation.

### 2.10. Fluorescence Lifetime Imaging Microscopy (FLIM)

**Figure 3:**
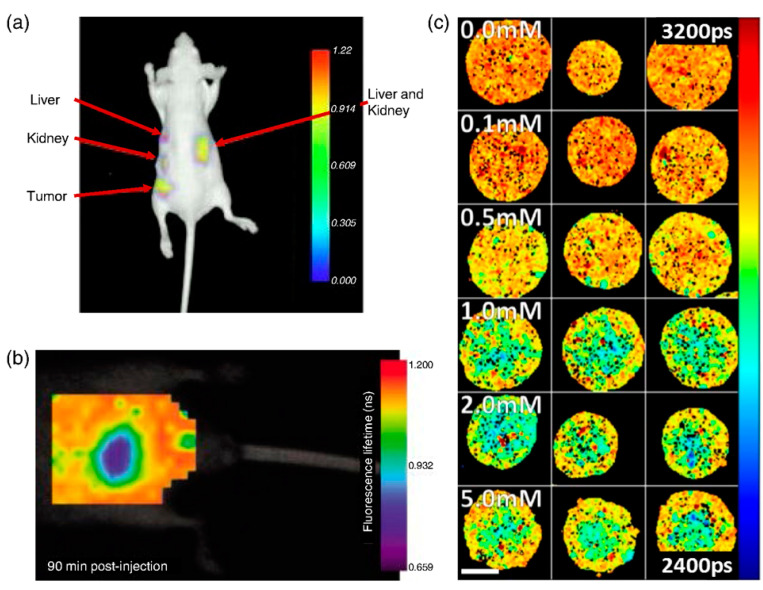
Fluorescence lifetime of mouse. The fluorescence lifetime image of A549-tumor in mouse and fluorescence lifetime of mouse after intravenous injection of LS-288 with the maps of the weighted mean fluorescence lifetime. (**a**) Fluorescence lifetime image of Cyp-GRD distributionat 24-h postinjection. (**b**) Fluorescence lifetime (heatmap) of mouse abdomen acquired 90 min after intravenous injection of LS-288. (**c**) FLIM maps of the weighted mean fluorescence lifetime. Reprinted with permission from reference [25], 2020, Datta et al., doi:10.1117/1.JBO.25.7.071203.

FLIM is a fundamental tool for biomedical imaging which provides high-resolution images of molecular contrast in living specimens. FLIM acquires not only morphological but also functional information about a tissue by measuring fluorophores’ lifetimes over time, which can be used to determine the state and malignancy of a sample [23]. It has applicability for both the research and diagnosis of the skin, brain, mouth, etc. [24]. It is more reliable than intensity-based approaches since fluorescence lifetime does not depend on concentration, sample absorption, or thickness [25]. In Figure 3, fluorescence lifetime imaging of mouse is seen. 

### 2.11. Vibrational Spectroscopy

In medical diagnostics, vibrational spectroscopy involving Raman spectroscopy and infrared spectroscopy (IR) are measurement techniques that may play a vital role. Raman spectroscopy analysis has the potential to extract medical and metabolic information [26]. It is also a highly promising technique in biological applications due to its lack of sensitivity to water [27]. IR spectroscopy uses the absorption of infrared light by molecular bonds to detect vibrations in the sample. The method is effective for investigating changes in the structure, function, and composition of tissues, cells, and biomolecules [28]. Fourier transform infrared spectroscopy (FTIR) is a form of IR spectroscopy for obtaining information about the presence of neoplasic, changes in biopsies, identifying bacteria, and types of arthritis [29]. Vibrational spectroscopy provides information on both cellular [30] and molecular [5] levels. These techniques are more often used in basic research [31], but they have tremendous potential in clinical contexts too. However, the measurements have to be further standardized before it can be routinely applied to clinical application.

### 2.12. Biomarkers

Biomarkers are a broad subcategory of medical signals that can be effectively observed or measured and whose accuracy and reproducibility can be measured [32]. Different types of biomarkers have clinical accountabilities in conducting treatment decisions and, depending on their subcategory, can be either diagnostic, prognostic, or predictive. The discovery of biomarkers is an important research task that involves many methods, such as proteomics and metabolomics [33]. In the scope of this manuscript, we only overview the usage of single biomarkers that are already routinely used in clinical applications, so they are represented as univariate data or multivariate data with a low number of variables. Combining different biomarkers in such a scope leads to only a slight increase in data dimensionality and can be studied via multivariate data analysis methods, rather than data fusion approaches. On the other side, combining biomarkers with image and spectral data is less trivial and is described in the respective subsections of Section 3.

## 3. Different Data Fusion Techniques

**Figure 4:**
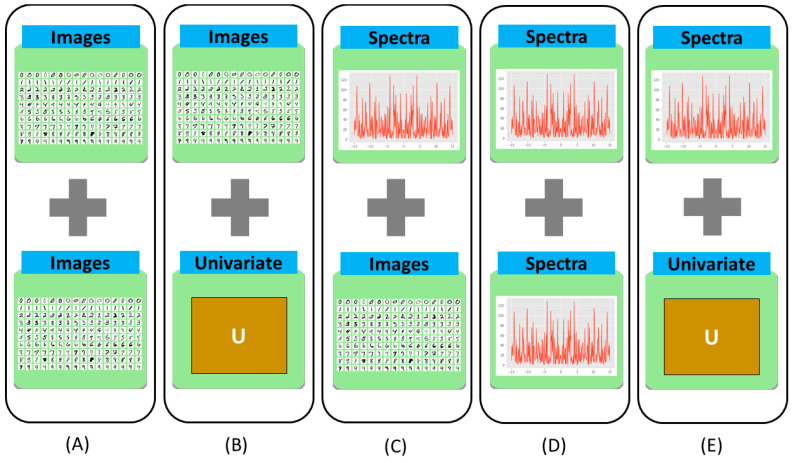
(**A**) Combination of image to image [Here, Image from MNIST [34] dataset]. (**B**) Combination of image to biomarker. (**C**) Combination of spectra to image. (**D**) Combinations of spectra to spectra. (**E**) Combination of spectra to biomarker.

Combining multiple data sources using data fusion schemes, aims to improve the extraction of the information from the different modalities and to increase the reliability of the interpretation. A data fusion can decrease prediction errors and increase the reliability of the results. The data fusion schemes being discussed in this study have their advantages and disadvantages. In the following Figure 4, we review a combination of multivariate data of different dimensionality orders and univariate data where we explore how the data are combined pairwise.

### 3.1. Data Fusion of Image Data

**Figure 5:**
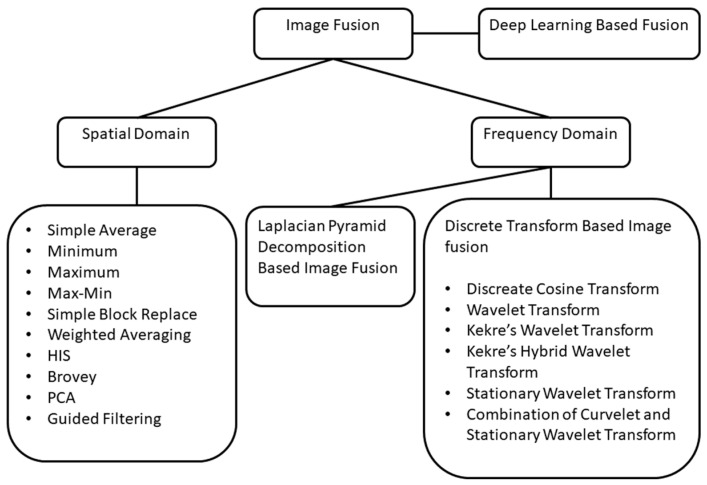
Image fusion techniques. Image fusion approaches with spatial and frequency domains indicate a variety of techniques and algorithms. Adapted with permission from reference [35], 2021, Springer Nature.

Researchers use data fusion techniques to achieve better outcomes in detecting different diseases as multiple modalities can contribute more sufficiently than a single modality. Images can be combined in multiple ways: deep learning-based function, spatial domain, or frequency domain, for instance. One of the image data fusion goals is to create images that are more understandable to humans and machines alike. In Figure 5, we can see how different image fusion techniques are used.

**Figure 6:**
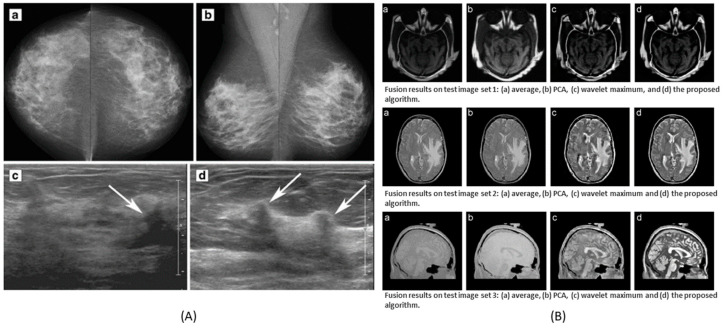
Data fusion as a result of different types of images. (**A**) Ultrasound and mammogram image fusion to detect breast cancer. (**B**) CT and MRI image fusion to provide high spatial quality of anatomical information and functional details of the diseases. (**A**) is reprinted with permissions from reference [36]. (**B**) is adapted with permissions from reference [37], 2008, Elsevier.

The combination of multivariate data of the second order of dimensionality (e.g., image data) can be seen from Figure 4A. Different types of medical images of human cells and organs indicate different kinds of details and features. Several combinations, e.g., ultrasound with mammogram, CT with MRI, and many more combinations perform well operating on different fusion levels, such as pixel-level, feature-level, and decision-level data fusion. Examples of some combinations can be seen in Figure 6.

In the context of medical diagnosis, there are many types of research that investigate image data fusion. Mishra et al. [38] studied a pixel-level fusion, where they applied a wavelet-based method and measured the fusion performance with a root mean square error (RMSE) and peak signal-to-noise ratio (PSNR) parameters. This technique yields promising results in terms of a lower RMSE and higher PSNR. Muzammil et al. [39] also described an approach on the same types of images utilizing a different method, the convolutional sparse image decomposition, at the pixel level. In another study, Tamilselvan et al. [40] combined pixel-level fusion with transform-based fusion and pyramid-based fusion employing MRI and CT images. They used multiple parameters, and based on the tentative results, some parameters, such as the mean difference, standard deviation, average difference, and RMSE were the smallest, while the entropy, PSNR, and mutual information were the largest. According to their experiment, they mentioned that it is likely to have an improved outcome when using a dual-tree complex wavelet transform method to diagnose MRI and CT images. 

From the previous discussion, we see that there are many investigations on pixel-level fusion using MRI and CT images but there are some studies also that investigated feature-level fusion over the same types of data. Rajkumar et al. [41] explained a feature-fusion technique for more accurate pathological information from MRI and CT images and they applied contourlet transform and redundancy discrete wavelet transform algorithms; according to the result of the investigation, they noticed that the redundancy discrete wavelet transform algorithm provides adequate information using an entropy metric and the contourlet transform performed great in terms of overall cross-entropy metrics. It is obvious to mention that there have been more studies on combining CT and MRI images. A weighted average fusion technique on MRI and CT images was applied by Agrawal et al. [42]. Their study included the dual-tree complex wavelet transform method and they proposed an innovative technique for estimating the parameters of the wavelet. According to them, a new method of fusion using convolutions of meridian distributions in the wavelet domain provided the best results compared to other methods. There is another research study on MRI and CT images where Nandeesh et al. [43] compared a fusion strategy using curvelet transform, discrete wavelet transform, principal component analysis, and stationary wavelet transform techniques. The performance of fusion was evaluated with the RMSE, entropy, PSNR, and mutual information. The output of the evaluation indicated that the curvelet transform method improves fusion performance as the curvelet transform can discover features from the direction of edges and has a great ability to analyze and track crucial image attributes. Kavitha et al. [44] also investigated a fusion technique to improve the image content by fusing images such as CT and MRI images to provide precise information to the doctor and clinical treatment. The images were decomposed using integer wavelet transforms and then the wavelet coefficients were fused using a neuro-fuzzy algorithm 

**Figure 7:**
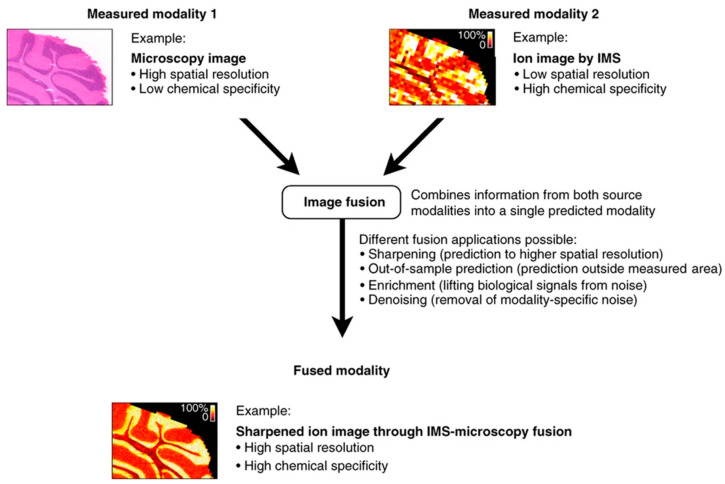
Fusion of microscopy and imaging mass spectrometry (IMS) to deliver microscopy’s spatial resolution and chemical specificity of IMS, that is combining two different modalities to make more accurate decision as single predicted modality. Reprinted with permission from reference [51], 2015, Springer Nature.

Many authors also paid attention to fusing MRI and SPECT data. Tan et al. [45] proposed an image fusion approach using a neural network fusion strategy where they tested the fusion result using MRI and SPECT. Other than CT, MRI, SPECT, and PET, there has been research on fusing other image data too. To mention some of them, a feature-level fusion technique was offered with combining pairs of magnetic resonance imaging such as T1, T2, and proton density brain images by Singh et al. [46] and to overcome the shift variance problem of discrete wavelet transforms, they used redundant discrete wavelet transforms. Adali et al. [47] also employed a similar fusion strategy with transposed independent vector analysis and the joint independent component analysis methods to fuse data from electroencephalography, structural MRI, and functional MRI. Along with the mentioned combinations of data fusion, according to the studies, Raman spectra, MALDI-IMS, microscopy, mass spectrometry, etc. are also popular measurement techniques in the field of data fusion to achieve precise information about diseases. Bedia et al. [48] illustrated an image fusion method in the combination of MALDI, infrared microscopy, and RGB images. They analyzed these fused images implementing a multivariate curve resolution alternating least squares (MCR-ALS) technique into biological tissue analysis. Piqueras et al. [49] also found MCR-ALS to be a better method for simplifying external spectral and spatial information and they tested their investigation of image fusion on different combinations such as MIR with Raman and MSI with Raman data. Accounting for the multi-component and global sparse representations of source images, Liu et al. [50] also described a pixel-level medical image fusion technique using convolutional sparsity analysis. Through different combinations of microscopy and IMS, Van de Plas et al. [51] described an imaging modality that predicts the molecular distribution of tissue samples. They used a partial least-squares (PLS) technique to model the distribution of measured values from both images when spatially paired. Figure 7 illustrates how cross-modalities can be applied to tissue samples using mass spectrometry and microscopy data. 

**Figure 8:**
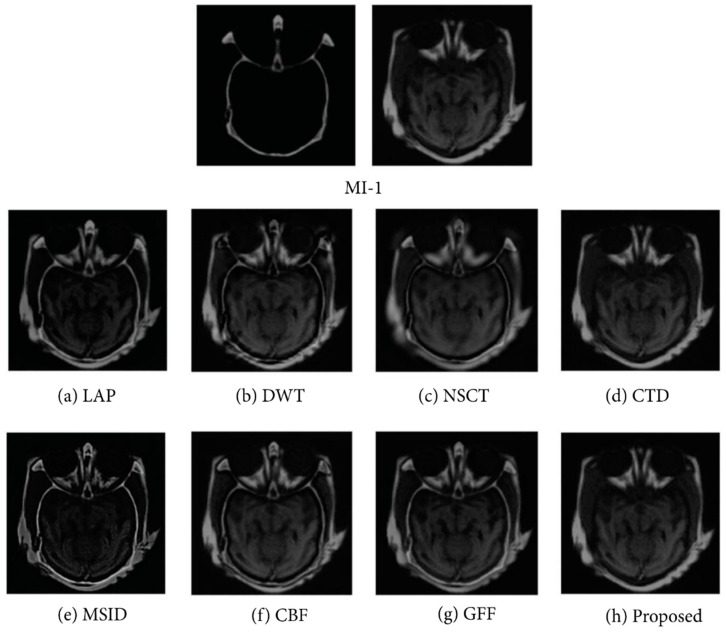
Outcome of the experiment “MI-1” was acquired using different fusion methods along with the proposed technique, e.g., decomposition, construction of decision map, sub-fusion of different layers, and image reconstruction. There are numerous traditional fusion methods based on mutual information levels and their associated enlarged regions. Reprinted with permission from reference [53], 2017, Zhang et al.

In data fusion, the combination of images and edges are also seen in a similar way as the image data fusion in Figure 4A. To combine the edge characteristics of sub-images, Wang et al. [52] presented a study to conduct pixel-level image fusion by using wavelet transformation algorithms to integrate multi-modal images and compared the fusion effect on MRI and PET images. The comparison experiments were conducted using three methods: high-pass filtering, weighted averaging, and traditional wavelet fusion. Moreover, according to the explanation of the feature-based data fusion approach which was proposed by Zhang et al. [53], they decomposed images from the source into two layers, detail layers and base layers, employing a local binary pattern method to obtain features in low-levels. Using saliency detection, the detail and base layers of the low-level features were used to construct weight maps. The map was adjusted by the fusion of both layers for continuing spatial stability among their corresponding layers and the images from the source. Then, the final output fused image was created by recombining mentioned layers appending the Laplacian pyramid, the discrete wavelet transforms, and the non-subsampled contourlet algorithms. In the investigation, they used nine pairs of medical images, e.g., MI-1, MI-2, MI-9 in testing image sets. The result of the testing image set MI-1 applying different algorithms with their proposed method is demonstrated in Figure 8.

Image fusion techniques have been advanced with the invention of hybrid methods. Hybrid imaging is the fusion of multiple imaging modalities to generate a new technique. A new and powerful modality comes into force when the advantages of the fused imaging technologies are combined. The hybrid approach improved diagnostic precision and lowered radiation exposure. Vitor et al. [54] studied that, in comparison to well-established PET/CT, PET/MRI offers a number of benefits, including excellent contrast and resolution and less ionizing radiation. PET/MRI is thus a potential technique for oncologic imaging of several locations, including the brain, head, neck, and liver. In another study, Sandulescu et al. [55] investigated the combination of anatomic and molecular imaging modalities. They mentioned real-time virtual sonography that combines ultrasound to contrast-enhanced CT/MRI. However, hybrid methods have some limitations as well. Most of the information offered by PET and SPECT is functional and may not always be immediately related to anatomical structures that are clearly identified. A significant drawback of these imaging methods is the absence of high-contrast anatomical information in the SPECT or PET data [56].

In general, the fusion of images aims to combine information from different imaging techniques applied to the same sample. The fused image will be more efficient in delivering information and for further image processing techniques. However, the images fused at low levels may produce spatial distortion, which is a negative factor for further processing. The low-level image data fusion requires precise co-registration, which is especially challenging if any manipulations with the sample are required between the measurements. 

### 3.2. Deep Learning in Data Fusion

**Figure 9:**
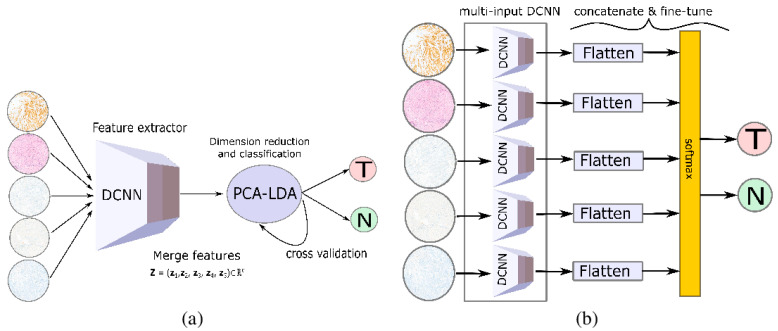
Data fusion approaches using transfer learning strategy. (**a**) Using a pre-trained DCNN as feature extractor prior to classic machine learning. (**b**) Multi-input DCNN with pre-trained network used as feature-extraction layers. Reprinted with permission from reference [62], 2021, Pradhan et al.

A wide use of deep learning techniques is noticed while investigating different methods of data fusion over multiple measurement techniques. In this section, we will discuss the applications of deep learning with a combination that is parallel to Figure 4A. A representative architecture described by Gao et al. [57] provides a good summary of the deep learning data fusion models. They mentioned different networks in their reviews such as a sparse autoencoder, convolutional neural network, deep belief network, and recurrent neural network. They note that the models are still in the preliminary stages, so challenges remain. They named redundant parameters as the first challenge of deep learning models that leads to large time consumption during training on large datasets. The second challenge was that all the semantic information in the multimodal data cannot be captured by the multimodal deep learning models, and the inconsistent data distribution was mentioned as the third challenge. In a different study, Huang et al. [58] investigated pixel-based data fusion where they combined electronic health records and medical imaging using a deep learning technique and mentioned that training a different deep learning model takes too long, and thus is insufficient for online multimodal data applications. Another pixel-based image fusion approach incorporating a deep learning method into the process was explained by Rajalingam et al. [59] for the fusion of multimodal medical images such as MRI, CT, and PET. They have used a convolutional neural network and claimed faster data processing and optimal visualization as the result of their investigations. Guo et al. [60] suggested a feature fusion-based deep learning network using eight sets of images, resulting in image quality, speed, and computing power. For the fusion, their proposed Hahn-PCNN-CNN network stated various issues such as image quality, handling power, and speed as these are still the concern of all investigators. The suggested network consisted of three modules: extraction of features, the fusion of features, and image modernization. In addition to the above fusion techniques, Iqbal et al. [61] developed an image fusion method, and they used two different CNN models and a long short-term memory (LSTM) when working with four modalities of MRI images to identify brain tumor delineation. As a result of their methodologies, the individual accuracy score for ConvNet was found to be 75%, the LSTM-based network was 80%, and ensemble fusion was 82.29% accurate. As part of further investigation, deep CNN and two transfer learning algorithms were utilized by Pradhan et al. [62] in a data fusion strategy for combining histology and IHC-stained images. The strategy offers numerous features of tissue-related relapses of breast cancer and the disease stage as well. Figure 9 demonstrates the data fusion approach using transfer learning with images of five stains as inputs to a pre-trained model. 

A CNN-based image data fusion method can effectively combine local features. However, this model fails to take the presence of long-range dependencies in the images into account [63]. One approach to overcome such a limitation is a transformer—a model with a self-attention mechanism that can weigh inputs according to their importance. Vibashan et al. [64] developed the transformer-based multi-scale fusion strategy that takes into account both local and global contexts. They trained an auto-encoder to extract deep features at various scales, then fused those multi-scale features utilizing a spatio-transformer fusion strategy. The advantage of such a combination is that the local features captured by CNN and the remote semantic and global information captured by transformers are complementary to each other. Wang et al. [65] studied the benefits of CNN and transformer combination in order to completely utilize both the global and the local information for improving the segmentation of 3D medical images. Applying deep learning-based data fusion strategies to biomedical data, researchers face issues that are generally common for such an approach as well as the issues specific to the analyzed data types and tasks. Stahlschmidt et al. [66] reviewed the issues that generally arise when analyzing biological data using multimodal deep learning techniques. They discuss issues related to insufficient data quantity, low quality, temporal characteristics (missing data), and poor model interpretability. As a possible solution, they suggest joint representation learning as preferable approach to make the interactivity of different levels of biomedical data and transfer learning overcome the sample size limitations.

### 3.3. Data Fusion of Image and Biomarker Data

The use of biomarkers in the research of cancer and development of drugs is crucial, and they are frequently used in clinical trials. Both imaging data and biomarkers are used routinely in the clinical context, but even when used together, they are typically processed separately and then interpreted by physicians. Alternatively, second-order multivariate (image) data and univariate data (biomarker, other clinical information, etc.) can be combined (see Figure 4B) using high-level data fusion approaches. While investigating data fusion, Metsis et al. [67] discussed how to use gene expression and MRS data for brain tumor classification. They used naive bayes for metabolites and a combination of information gain and wrapper features for genes and obtained the highest accuracy 87.23%. In another study, to measure lung cancer risk, Gong et al. [68] used serum biomarkers that were extracted from samples of blood and CT images. For the segmentation of lung nodules using a computer-aided diagnosis, they used a four-step segmentation method and computed 78 imaging features from each nodule segment of CT images. The serum biomarkers were used along with features of the CT images to build two support vector machine (SVM) classifiers. The obtained results were validated by oversampling, relief feature selection, and leave-one-out cross validation of the SVM classifiers on an overall dataset. They used an information-fusion method for combining the predictions generated by the two SVM classifiers with a focus on the improvement of computer aided diagnoses; the method includes the minimum, maximum, and weighting average data fusion. By the same token, Fu et al. [69] used multimodal information fusion models to combine serum biomarkers with 3D lung CT images to analyze the types of pulmonary nodules: squamous cell carcinoma, adenocarcinoma, inflammation, and benign. 

### 3.4. Data Fusion of Spectra Data

In both the medical and biomedical fields, hyperspectral imaging (HSI), which provides spectral and spatial information about samples at the same time, is receiving increasing interest. Chemical components can be effectively represented by spectral information and the spatial information can reveal the sample structure. Thus, the combination of spectral and spatial information can improve the reliability of the disease analysis. Figure 4C represents the combination of multivariate data of the first and second order. In the data fusion context, there have been investigations on the combination of spectra with other imaging modalities. Neumann et al. [70] explored (IR) Infrared spectroscopic imaging and MALDI-IMS with a range of IR absorption bands where chemical sharpening of MS images was possible. Thus, the optimum sharpening of ion images with minimal artifacts was achieved. They were able to identify distributions of lipid, a functionally essential, morphologically, and chemically complex tissue of the brain, thanks to the intrinsic spatial agreement between these modalities. They employed the mid-level data fusion approach for integrating information of chemicals and increasing the ability to distinguish structurally relevant regions with k-means clustering. Because MALDI-IMS is a method of ionization, there was no laser damage in the infrared images, allowing typical staining methods. Another study by Attia et al. [71] described a method for monitoring the response to infection by combining MALDI-IMS and MRI. Therefore, the combination of MALDI-IMS and MRI allowed for the study of inflammation during infection for a biological approach. Research in the medical and biomedical aspects shows that the tissue samples are used in scientific research to detect disease progression, drugs development, and improvement in medical care. To integrate Raman micro spectroscopic imaging and (MALDI-IMS) matrix-assisted laser desorption/ionization mass spectrometric imaging for tissue-based research, Bocklitz et al. [72] built a computational approach where their results confirmed a spectral histopathology based on Raman spectroscopy with MALDI-IMS and within the Raman measured region. They used a PCA-LDA model to train tissue groups annotated by the pathologist. Further tissue-based research was conducted by Rabe et al. [73] where they presented an integrated approach using MALDI-MSI and a non-destructive Fourier transform infrared based on FTIR imaging for multimodal tissue analysis; their findings indicated that MSI data acquisition and interpretation can be automatically guided by FTIR microscopy without the need of a previous annotation of histopathological tissue. The combination of MALDI-IMS with confocal Raman microscopy is an integral part of data fusion research. An innovative approach of correlated imaging combining MALDI-IMS and confocal Raman microscopy was introduced by Ahlf et al. [74] to investigate cell cultures of the operational and chemical mixture inherent in 3D. The most chemically informative elements were identified using principal component analysis and they were subsequently integrated using the correlation of digital images. Each procedure of the primary components was shown using the method, allowing them to be compared on comparable scales of spatial length. 

Moreover, it is seen from the impact of data fusion investigations that, the data fusions strategy plays a great role also in basic skin research, clinical dermatology, pharmacology, and cosmetic research, as well as non-invasive blood detection. The study analyzed by Caspers et al. [75] demonstrates the utilization of vivo confocal Raman spectroscopy and confocal microscopy in conjunction with confocal scanning laser microscopy (CSLM). Chen et al. [76] reported Raman and infrared Fourier spectroscopy diagnosis of thyroid dysfunction in serum where they used a pattern recognition algorithm and PCA as the best analytical model. According to them, the spectral fusion accuracy, particular infrared spectral accuracy, and Raman spectral accuracy of SVM were 83.48%, 80%, and 78.26%, respectively. Furthermore, research based on optical coherence tomography and Raman spectroscopy was explained by Rangaraju et al. [77] and they have developed significant efficacy in ex vivo skin, facilitating evaluation of the potential of coupled Raman spectroscopy and optical coherence tomography in vivo models. The combined efficacy of RSOCT reported an overall mean accuracy of 85% and ROCAUC = 0.94 in identifying injured wounds. Furthermore, Placzek et al. [78] explained the combination of the same measurement technique for bladder cancer diagnosis, where the molecular and morphological features from the same location were acquired with co-registration, thus allowing them to be correlated. Besides, in an investigation by Schie et al. [79], it is noticed that a combination of Raman spectroscopy with different optical methods such as coherent anti-Stokes Raman scattering microscopy, second-harmonic generation, autofluorescence microscopy, stimulated Raman scattering (CARS) microscopy, spectroscopy, fluorescence lifetime imaging, and others improve diagnostic performance and target cardiovascular disease, inflammatory disease, and cancer.

**Figure 10:**
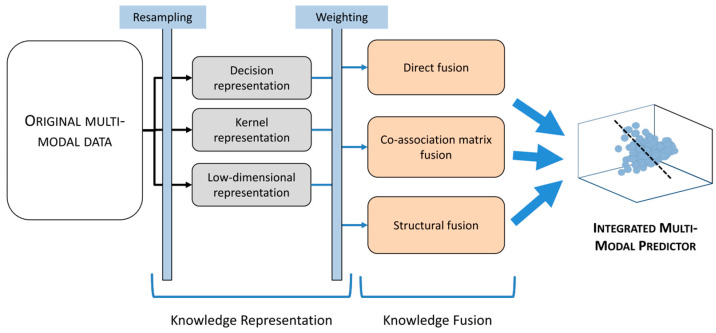
Modality-specific scale and dimension differences are eliminated through knowledge representation, which transforms each modality individually into a unified space. Resampling refers to the generation of multiple representations and weighting considers individual characteristics of the modalities. In order to achieve the best fused result from different modalities, complementary information from every channel should be used. Reprinted with permission from reference [81], 2018, Viswanath et al.

To execute the data fusion strategy, these three kinds of data: fluorescence spectroscopy, nuclear magnetic resonance spectroscopy, and liquid chromatography mass spectrometry were framed as a tensor factorization and coupled matrix issue by Acar et al. [80]. They discovered that utilizing the structure of low rank and higher-order data collections, tensor factorization and the coupled matrix model may capture the basic factors successfully during the period of structure revealing. The preliminary data revealed that there were unshared and shared components, achieving 71.4% accuracy with some of the shared factors in differentiating the two groups (with 63.6% sensitivity and 78.1% specificity). Satish E. Viswanath et al. [81] investigated radiomics features from T2w MRI in vivo, protein mass spectrometry features with histomorphometry features from histopathology, and volumetric measurements from MRI and suggested that using kernel representations with dimensionality reduction-based fusion could be the most successful. In Figure 10, the steps for dimensionality reduction-based multimodal data fusion are visualized. In addition to the further research on fusion strategy, the pathophysiology of the brain was evaluated by Porta Siegel et al. [82] using MRI and MSI to establish the correlation between ex and in vivo molecular imaging modality. The modulation of insignificant peptides and endogenous metabolites was assessed according to the disease status. In the MSI community, it is observed that one of the major challenges is managing and interpreting big data. 

**Figure 11:**
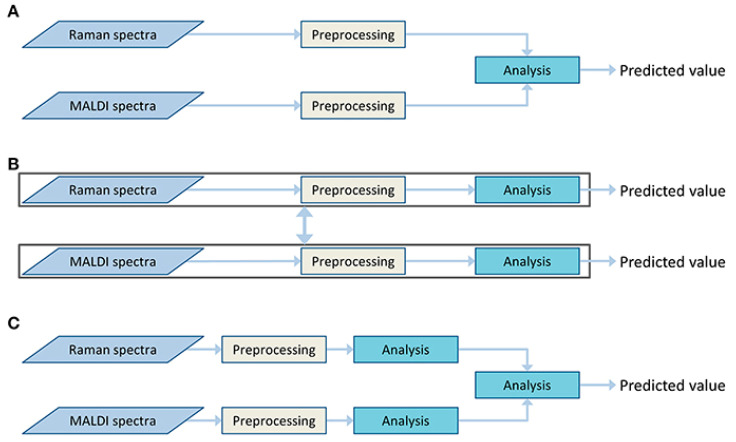
Different types of fusion architectures (**A**) Centralized, in which both types of data are analyzed together after pre-processing. (**B**) Decentralized, where both types of data are analyzed and predicted individually. (**C**) Distributed, where both types of data are analyzed independently and after that both outputs of analysis are analyzed again and a prediction is made. Reprinted with permission from reference [84], 2021, Ryabchykov et al.

From the discussion above, we observe multiple fusion techniques over the combinations of spectra data with different modalities. There are also lots of studies that include fusion techniques on the combination of spectra and spectra data that are depicted in Figure 4D. First, Bocklitz et al. [83] used two techniques for a mouse brain with MALDI-TOF and Raman imaging. The use and interpretation of complementary data were possible when both techniques were used together. They also showed how to interpret Raman spectra using spectrum information from MALDI-IMS studies. Similarly, Ryabchykov et al. [84] investigated a data fusion strategy to combine Raman spectroscopy with MALDI-IMS data. They showed that in order to uncover more spectral information, the weighting of data was required in the fusion of the data. Weighting techniques were evaluated by assessing the data variance described by PCA and visualizing the PCA findings for each of the data types and the combination of the data. In Figure 11, we can see the type of fusion architecture that they proposed.

Generally, there are different approaches in data fusion of image to spectra. There might be a combination of one image to multiple spectra and for multiple images to one spectrum. In this case, high-level and mid-level data fusions become more convenient for image-to-spectra data fusion. 

### 3.5. Data Fusion of Spectra and Biomarker Data

The combination of multivariate data of the first order (spectra) and the univariate data of the zero order (biomarker, other clinical information, etc.) has been depicted in Figure 4E. An analysis of leukocytes can show whether the host is responding to specific viruses or is dysregulated in cases of sepsis. Ramoji et al. [85] used Raman spectroscopy to describe individual peripheral blood leukocytes in order to diagnose sepsis and infections and they utilized known clinical scores, biomarkers, and blood counts as reference diagnoses. Based on Raman data binary classification models, they were able to discriminate between the infected and non-infected patients as well as patients with or without sepsis with accuracies comparable to those attained with recognized biomarkers. For an initial data review and to depict the spectral separation of the clusters, logistic regression analysis was utilized by them. In terms of spectra and biomarker combination, Bro et al. [86] reported that combining biomarkers with fluorescence spectroscopy from plasma samples ensures the capacities for the prompt diagnosis of colorectal cancer rather than a single marker. Hence, data fusion is important to identify diseases with different modalities. Spectroscopic measures are usually delivering information on the cellular or molecular level. In the researched in vitro experiments, biomarkers may also deliver the information about the specific cellular response, which can then be effectively combined with the spectral data. The situation is rather different in the context of in vivo experiments or in the clinical context. It is possible to receive an overview in the form of two different perspectives of two different levels when spectroscopic techniques that provide information on the molecular level are combined with biomarkers that provide information about the patient in general. 

## 4. Summary and Outlook

In medical and biomedical diagnostics, data fusion techniques combine multiple types of data for predictive tasks such as disease detection. Thus, data fusion combines different data types/modalities and enhances the efficiency and accuracy of a prediction with respect to the prediction using a single modality. Researchers use various data fusion methods that act on different levels: pixel-level, feature-level, and decision-level. With these data fusion techniques, information can be fused on different levels and different information represented by different data can be used at the same time. Beside classical machine learning, deep learning methods also gained significant popularity in data fusion investigations because they facilitate a flexible fusing and provide promising results. Besides, the fusion of data encounters numerous methodological challenges. The major unresolved challenge of data fusion in the biomedical area is limited data. In order to overcome the challenge of limited data, we need models that can be utilized with small training datasets. One possible way of resolving the data-size issue can be addressed by sophisticated augmentation strategies. It is also possible to reduce the complexity of data-by-data standardization and the dimensionality reduction that can be handled by data pre-processing and feature engineering, selection, or extraction. Another challenge is the development of sample-size-planning (SSP) approaches suitable for multivariate and image data. SSP is not trivial even for multivariate data [87], but in the case of combining different data types, the complexity of the task increases even further. Additionally, data conflict can be introduced in different data fusion schemes and sincere attention is needed to face the challenges in data fusion. These challenges can be addressed with data adjustment, data association, and data structure preparation. Nevertheless, these challenging circumstances are acceptable because a comprehensive view of the sample can be gained.

This review paper presents studies on data fusion for different measurement techniques and imaging approaches including ultrasonography, single photon emission computed tomography, positron emission tomography, magnetic resonance imaging, computed tomography, Raman spectroscopy, MALDI-IMS, mammography, and fluorescence lifetime imaging microscopy. As the capability of data fusion is huge and depends on the task and data types involved, systematic studies are needed. These systematic studies should clarify which data fusion scheme, data fusion levels, and machine learning methods are optimally suited to solve a given task. Additionally, larger datasets are needed to investigate data fusion techniques and improve them. Besides the challenges, the gains achieved by data fusion, e.g., the usage of different information to describe samples comprehensively, are perfect for improving data-driven models in biomedical tasks.
